# Evaluation of an Ambient Artificial Intelligence Documentation Platform for Clinicians

**DOI:** 10.1001/jamanetworkopen.2025.8614

**Published:** 2025-05-02

**Authors:** Cheryl D. Stults, Sien Deng, Meghan C. Martinez, Joseph Wilcox, Nina Szwerinski, Kevin H. Chen, Stephanie Driscoll, Joanna Washburn, Veena G. Jones

**Affiliations:** 1 Sutter Health, Palo Alto, California

## Abstract

**Importance:**

The increase of electronic health record (EHR) work negatively impacts clinician well-being. One potential solution is incorporating an ambient artificial intelligence (AI) documentation platform.

**Objective:**

To understand clinician experience before and after implementing ambient AI.

**Design, Setting, and Participants:**

This quality improvement study was a pilot evaluation with before and after survey and EHR metrics conducted at a large health care organization in Northern and Central California. Clinicians were purposively sampled to be representative of region and specialty. Ambient AI was implemented in April 2024 with EHR data from 3 months before and after implementation. Data were analyzed from May to September 2024.

**Exposure:**

Ambient AI access.

**Main Outcomes and Measures:**

Metrics of time were examined in notes per appointment, off-hour EHR activities (5:30 pm to 7:00 am on weekdays and nonscheduled weekends and holidays), documentation note length, progress note length, NASA Task Load Index (NASA-TLX) score, mini-*Z* burnout question, and overall experience. It was hypothesized that time in notes per appointment would decrease and clinical well-being would improve. Logistic regression and linear mixed-effect models were used.

**Results:**

Among 100 clinicians (53 male [53.0%]; mean [SD] age, 48.9 [11.0] years), 58 clinicians (58.0%) were in primary care and 92 clinicians had EHR metrics. Among 57 clinicians who completed both preimplementation and postimplementation surveys, there was a decrease in burnout from 24 clinicians (42.1%) to 20 clinicians (35.1%), although this was not a significant difference (*P* = .12). Mean (SD) NASA-TLX scores all decreased after using ambient AI: mental demand of note writing (12.2 [4.0] to 6.3 [3.7]), hurried or rushed pace (13.2 [4.0] to 6.4 [4.2]), and effort to accomplish note writing (12.5 [4.1] to 7.4 [4.3]) (all *P* < .001). Mean (SD) time in notes per appointment significantly decreased from 6.2 (4.0) to 5.3 (3.5) minutes (*P* < .001), with a bigger decrease for female vs male clinicians (8.1 [3.9] to 6.7 [3.6] minutes vs 4.7 [3.5] to 4.2 [3.1] minutes; *P* = .001). More primary care clinicians (33 of 38 clinicians [85.8%]) reported that ambient AI improved overall satisfaction at work compared with clinicians in medical (4 of 11 clinicians [36.4%]) and surgical (4 of 8 clinicians [50.0%]) subspecialties (*P* < .001). After adjusting for participant characteristics, model results suggested that mean scores for NASA-TLX decreased for mental demand (−6.12 [95% CI, −7.52 to −4.72]), hurried or rushed pace (−6.96 [95% CI, −8.42 to −5.50]), and effort to accomplish note writing (−5.57 [95% CI, −6.93 to −4.21]), while mean time in note taking decreased by less than 1 minute per appointment (0.91 minutes [95% CI, −1.20 to −0.62 minutes]) (all *P* < .001).

**Conclusions and Relevance:**

This study found that ambient AI was associated with improved overall experience and time in notes for clinicians but with varying outcomes by sex and specialty. Future research should investigate outcomes after widescale expansion of this rapidly evolving technology.

## Key Points

**Question:**

Is an ambient artificial intelligence (AI) documentation platform associated with clinician documentation time and well-being?

**Findings:**

In this quality improvement study at a single health care organization using before and after survey results and electronic health record data, ambient AI was associated with decreased time in notes per appointment. Ambient AI was associated with improved clinician satisfaction at work and decreased cognitive load.

**Meaning:**

These results suggest that ambient AI may be a potential solution to improve the experience of work for clinicians by decreasing the burden of clinical documentation.

## Teaser

This quality improvement study investigates the association of an ambient artificial intelligence documentation platform with clinician work time and well-being.

## Introduction

Clinician burnout has increased^1^ and overall well-being has declined among physicians and advanced practice clinicians^2,3^ in part because of increasing electronic health record (EHR) work, especially outside regular clinic working hours.^4^ Studies suggest that clinicians spend 2 hours documenting for every hour spent with patients,^5^ and a recent national survey found that 77.42% of clinicians reported that excessive documentation tasks led to longer clinic hours or the need to work from home.^4^ However, another study found that clinicians who were very satisfied with the EHR system had a decreased likelihood of burnout,^6^ suggesting that improvements to the EHR experience could alleviate burnout.

Tools aiding clinicians with documentation include in-person and virtual scribes and, more recently, ambient artificial intelligence listening programs (ambient AI). Ambient AI uses generative AI to listen to a clinical visit and generate a progress note for clinician review.^7,8,9^ Initial survey results showed significant improvements in self-reported documentation aspects, such as a reduction in task load,^10^ ease of processes,^8^ improved clinician wellness (including overall well-being^8^ and decreased burnout), and a perceived impact on patient experience^8^ after ambient AI. However, the association of ambient AI with EHR metrics has been mixed, with 1 study finding that use of ambient AI was associated with reductions in time spent in notes during appointments but no difference in off-hour EHR activities (5:30 PM to 7:00 AM on weekdays and nonscheduled weekends and holidays)^11^ while another study^12^ did not find significant differences for time in notes or work time outside of work.

Sutter Health recently piloted an ambient AI platform to support clinician documentation. It was hypothesized that ambient AI would be associated with reduced clinician time documenting and improved well-being from decreased time in the EHR.

## Methods

This evaluation was determined to be a quality improvement study by the Sutter Health Institutional Review Board. We followed the Standards for Quality Improvement Reporting Excellence (SQUIRE) reporting guideline. Sutter Health is a large, nonprofit, multispecialty integrated health care delivery system spanning 21 counties across Northern and Central California using a single instance of the Epic EHR system. A total of 100 ambulatory clinicians were specifically selected to use the ambient AI platform Abridge (Abridge AI, Inc), with proportional geographic representation of the main regions, representation from primary care and numerous specialties, and clinical leaders or medical informatics champions.

### Surveys

Prior to the platform’s launch on April 10, 2024, participants were asked to complete a preimplementation survey with questions about burnout (mini-*Z* burnout question from the American Medical Association); task load for mental demand, temporal demand, and effort from the NASA Task Load Index (NASA-TLX)^13^; time spent per week completing notes outside of clinic hours; and ability to provide undivided attention to patients during a visit. The postimplementation survey included these same questions and additional questions about the overall experience, whether the clinician would recommend Abridge to others (scale of 0-10), note quality, and work satisfaction, with 2 open-ended questions about ambient AI impact and suggestions for improvements. Answering surveys served as implied consent to participate. To maintain a full 100-person cohort, we reassigned licenses to a new clinician for any individuals who actively withdrew from the pilot project or did not use ambient AI for most of the 30 days. These added clinicians were given preparticipation and postparticipation surveys.

We included only clinicians who completed both preintervention and postintervention surveys. Clinician demographics, including sex, race and ethnicity, specialty, role type, and clinical full-time equivalent, were obtained from the EHR and human resource databases. Race categories in databases were American Indian, Asian, Black, White, multiple races, and unknown. Ethnicity categories were Hispanic, non-Hispanic, and unknown. Categories without any respondents were omitted for this study. We included race and ethnicity to test for an association and control for this in multilevel modeling. We dichotomized Likert scale items by categorizing “agree or strongly agree” as *yes*, while all remaining responses were categorized as *no*. We categorized individuals selecting options 3, 4, or 5 as *burnout*. We dichotomized responses to “1 hour or less” and “more than 1 hour” for mean time spent note writing outside clinic hours.

### EHR Metrics

Outcomes associated with ambient AI were also assessed using the following EHR metrics: time in notes per appointment, off-hour EHR activities (an Epic metric known as *pajama time*), documentation length, and progress note length. All metrics were collected monthly for 3 months before implementation (preimplementation period) and 3 months after implementation (postimplementation period), and the mean over the preimplementation and postimplementation period was found for each participant.

### Statistical Analysis

Descriptive statistics summarized each outcome. Demographic characteristics of total respondents and those who responded to both preimplementation and postimplementation surveys were compared. We used the χ^2^ test or Fisher exact test for categorical variables and *t* test and Mann-Whitney *U* test for the mean and median of continuous variables. We used the McNemar test for dichotomous outcomes and paired *t* test and paired Wilcoxon signed-rank test to assess changes in the mean and median of continuous outcomes separately from before and after the intervention. We also assessed outcome changes for each sex and categorized specialty group (ie, primary care, medical subspecialties, and surgical subspecialties). Bonferroni adjustment was applied for multiple comparisons. We then used difference-in-difference (DiD) analysis to see whether ambient AI had varying associations by group. DiD compared outcome changes between groups before and after using ambient AI. Multivariate linear mixed models were used to estimate outcomes associated with ambient AI implementation, adjusting for participant characteristics and accounting for individual differences among participants, as well as exploring any associations between participant characteristics and outcomes. For questions only in the postimplementation survey, the χ^2^ test was used to assess group differences in binary outcomes, while 1-way analysis of variance and the Moon median test were separately used to test group differences in mean and median of continuous outcomes. Logistic regressions were conducted for binary outcomes, controlling for participant characteristics.

Practical thematic analysis^14^ of open-ended survey questions was done by a qualitative analyst (M.C.M.), who first created code categories based on responses. These code categories were then refined by another qualitative researcher (C.D.S.), who reviewed all coded data, made minor adjustments, and created themes. These themes have been summarized into those most frequently mentioned by respondents. We combined responses to the 2 free-response questions because similar code categories emerged across the data.

For EHR metrics, we calculated mean and median overall across participants and by sex and specialties. We used paired *t* test to compare the mean of metrics and paired Wilcoxon signed-rank test to compare the median of metrics in the preimplementation vs postimplementation period. DiD analysis was further used to compare changes in mean EHR metrics between groups before and after using ambient AI. Multivariate linear mixed models were conducted to estimate the association of ambient AI implementation with each EHR metric by adjusting for participant characteristics and accounting for individual differences among participants. All results are reported as 2-sided tests, and statistical significance was set at α = .05. Statistical analyses were conducted using R statistical software version 4.3.2 (R Project for Statistical Computing). Data were analyzed from May to September 2024.

## Results

The initial pilot project included 100 clinicians (53 male [53.0%]; mean [SD] age, 48.9 [11.0] years; 18 Asian [18.0%], 62 White [62.0%], and 3 multiracial [30%]; 8 Hispanic [8.0%]), and 57 clinicians completed both preimplementation and postimplementation surveys. Clinicians with responses to both surveys were similar to the overall group (Table 1), except for by specialty (primary care: 58 clinicians overall [58.0%] vs 38 clinicians with both surveys [66.7%]; surgical subspecialties: 21 clinicians overall [21.0%] vs 8 clinicians with both surveys [14.0%]) and the proportion with full-time equivalent less than 0.7 (38 clinicians overall [38.0%] vs 27 clinicians with both surveys [47.3%]). No significant differences were found between the overall clinician group and 57 respondents with both surveys in any characteristic.

**Table 1.** Characteristics of Clinicians

Characteristic	Clinicians, No. (%)		*P* value
	Total (n = 100)	With both surveys (n = 57)	
Sex			
Female	47 (47.0)	26 (45.6)	.87
Male	53 (53.0)	31 (54.4)	
Race^a^			
Asian	18 (18.0)	10 (17.5)	.96
White	62 (62.0)	37 (64.9)	
Multiple races	3 (3.0)	1 (1.8)	
Unknown	17 (17.0)	9 (15.8)	
Ethnicity^a^			
Hispanic	78 (78.0)	46 (80.7)	.81
Non-Hispanic	8 (8.0)	3 (5.3)	
Unknown	14 (14.0)	8 (14.0)	
Age, y^b^			
Mean (SD)	48.9 (11.0)	50.3 (10.8)	.48
Median (IQR)	48.5 (40-57)	51 (43-57)	.50
Clinician type			
Physician	92 (92.0)	53 (93.0)	.82
Advanced practice clinician^c^	8 (8.0)	4 (7.0)	
Specialty			
Primary care^d^	58 (58.0)	38 (66.7)	.48
Medical subspecialty^e^	21 (21.0)	11 (19.3)	
Surgical subspecialty^f^	21 (21.0)	8 (14.0)	
Years of service^g^			
Mean (SD)	11.4 (8.2)	12.7 (8.0)	.36
Median (IQR)	11 (5-16)	12 (7-16)	.29
Clinical FTE			
<0.5	10 (10.0)	8 (14.0)	.69
0.5 to <0.7	28 (28.0)	19 (33.3)	
0.7 to ≤1	46 (46.0)	23 (40.4)	
Unknown	16 (16.0)	7 (12.3)	

Abbreviation: FTE, full-time equivalent.

^a^ Race and ethnicity were obtained from the electronic health record and human resources.

^b^ Age was missing for 10 clinicians overall and 7 clinicians with both survey results.

^c^ Includes nurse practitioner and physician assistant.

^d^ Includes internal medicine, family medicine, pediatrics, and urgent care.

^e^ Includes neurology, hematology and oncology, cardiology, gastroenterology, rheumatology, dermatology, psychiatry, pulmonology, and endocrinology and metabolism.

^f^ Includes podiatric medicine and surgery, interventional cardiology, orthopedic surgery, otolaryngology, obstetrics and gynecology, neurosurgery, anesthesiology, plastic surgery, and urology.

^g^ Years of service was missing for 16 clinicians overall and 7 clinicians with both survey results.

### Preimplementation and Postimplementation Survey Analysis

Among clinicians with both surveys, we found a small decrease in burnout, from 24 clinicians (42.1%) to 20 clinicians (35.1%), although this difference was not statistically significant (*P* = .12) (Table 2). For giving patients their undivided attention, the proportion of clinicians who responded “agree/strongly agree” increased from 33 clinicians (57.9%) to 53 clinicians (93.0%) (*P* < .001). On a scale from 0 to 20, the following 3 items’ mean (SD) scores all decreased after ambient AI was used: mental demand (12.2 [4.0] to 6.3 [3.7]), hurried or rushed pace (13.2 [4.0] to 6.4 [4.2]), and effort to accomplish note writing (12.5 [4.1] to 7.4 [4.3]) (all *P* < .001). More respondents (31 clinicians [54.4%]) noted in the postimplementation survey that they spent 1 hour or less per week on notes compared with the preimplementation period (8 clinicians [14.0%]) (*P* < .001). All outcomes except burnout remained significant after Bonferroni correction (α = 0.05/6). For only postimplementation survey questions, 41 respondents (71.9%) agreed or strongly agreed that Abridge had increased their work satisfaction. Out of a maximum score of 10, the mean (SD) score was 7.8 (1.8) for overall experience and 8.5 (2.1) for recommend to others.

**Table 2.** Preimplementation and Postimplementation Survey Results

Variable	Overall (N = 57)			Male (n = 31)			Female (n = 26)			DiD *P* value^a^	Primary care (n = 38)			Medical subspecialties (n = 11)			DiD *P* value^b^	Surgical subspecialties (n = 8)			DiD *P* value^c^
	No. (%)		*P* value	No. (%)		*P* value	No. (%)		*P* value		No. (%)		*P* value	No. (%)		*P* value		No. (%)		*P* value	
	Pre	Post		Pre	Post		Pre	Post			Pre	Post		Pre	Post			Pre	Post		
Mental demand																					
Mean (SD)^d^	12.2 (4.0)	6.3 (3.7)	<.001	11.3 (4.1)	6.3 (3.3)	<.001	13.2 (3.6)	6.3 (4.2)	<.001	.16	12.5 (4.1)	6.0 (3.3)	<.001	12.9 (3.6)	8.2 (4.8)	.006	.29	9.6 (3.7)	5.3 (2.9)	.005	.26
Median (IQR)^e^	12 (10-15)	5 (4-10)	<.001	10 (8-15)	5 (5-8.5)	<.001	14 (10-15)	5 (2.25-10)	<.001		12.5 (10-15)	5 (4.25-7.75)	<.001	14 (10-15)	10 (4.5-11)	.02		10 (7.25-11)	5 (4.25-7)	.02	
Temporal demand																					
Mean (SD)^d^	13.2 (4.0)	6.4 (4.2)	<.001	13.3 (3.6)	6.2 (4.0)	<.001	13.1 (4.4)	6.7 (4.5)	<.001	.65	13.8 (3.7)	6.1 (4.0)	<.001	12.5 (4.9)	7.3 (4.1)	.009	.19	11.8 (3.8)	7.1 (5.4)	.01	.16
Median (IQR)^e^	13 (10-15)	5 (5-8)	<.001	15 (10-15)	5 (4-8)	<.001	12.5 (10-17.25)	5 (5-8.75)	<.001		15 (10-16.75)	5 (4.25-7)	<.001	14 (10-15)	8 (6-10)	.02		12 (10-12.75)	5 (4.25-10.5)	.14	
Effort																					
Mean (SD)^d^	12.5 (4.1)	7.4 (4.3)	<.001	11.5 (4.3)	7 (4.6)	<.001	13.6 (3.6)	7.9 (4)	.001	.40	12.6 (3.4)	6.6 (3.4)	<.001	13.8 (4.9)	9.5 (5.6)	.01	.33	10 (5.3)	8.1 (5.6)	.52	.04
Median (IQR)^e^	14 (10-15)	5 (5-10)	<.001	12 (9-15)	5 (5-10)	<.001	15 (12-15)	7 (5-10)	<.001		12 (10-15)	5 (5-8)	<.001	15 (11-16)	10 (5-13)	.02		10 (5-15)	7.5 (4.25-12.75)	.73	
Mini-*Z* burnout (selecting 3, 4, or 5)^f^	24 (42.1)	20 (35.1)	.12	8 (25.8)	7 (22.6)	.85	16 (61.5)	13 (50)	.71	.51	16 (42.1)	13 (34.2)	.21	6 (54.5)	5 (45.5)	1	.92	2 (25)	2 (25)	1	.66
Undivided attention (agree/strongly agree)^f^	33 (57.9)	53 (93.0)	<.001	19 (61.2)	28 (90.3)	.001	14 (53.8)	25 (96.2)	.002	.32	20 (52.6)	36 (94.7)	<.001	7 (63.6)	9 (81.8)	.64	.15	6 (75)	8 (100)	NA	.99
Note writing outside work (≤1 h)^f^	8 (14.0)	31 (54.4)	<.001	6 (19.4)	23 (74.2)	<.001	2 (7.7)	8 (30.8)	.008	.13	5 (13.2)	21 (55.3)	<.001	0 (0)	5 (54.5)	NA	.99	3 (37.5)	5 (62.5)	.62	.36

Abbreviation: DiD, difference in difference.

^a^ DiD for male vs female.

^b^ DiD for primary care vs medical subspecialties.

^c^ DiD for primary care vs surgical subspecialties.

^d^ Paired *t* test.

^e^ Wilcoxon signed-rank test.

^f^ McNemar test.

We conducted subgroup analyses by sex and specialty (Table 2). A consistent trend in preimplementation to postimplementation changes in outcomes was observed in each subgroup. No significant differences were found in preimplementation vs postimplementation changes or overall evaluation of ambient AI between males and females. A higher percentage of primary care participants indicated “agree/strongly agree” when asked if their work satisfaction has been increased by Abridge (33 of 38 clinicians [85.8%]) vs medical (4 of 11 clinicians [36.4%]) and surgical (4 of 8 clinicians [50.0%]) subspecialties (*P* < .001); primary care participants also had a higher mean (SD) score in overall experience (8.2 [1.1]) vs medical (6.6 [2.6]) and surgical (7.6 [2.6]) subspecialties (*P* = .04) and the recommend to others outcome (9.1 [1.1]) vs medical (6.9 [2.9]) and surgical (7.8 [3.1]) subspecialties (*P* = .003) (see eTable 1 in Supplement 1 for post-AI–only survey results). After Bonferroni adjustment for subgroup comparisons (α = 0.05/30), most outcomes remained significant; however, preimplementation to postimplementation changes in NASA-TLX questions became nonsignificant for medical and surgical subspecialties. Adjusting for survey respondent characteristics, linear mixed modeling (eTable 2 in Supplement 1) indicated a decrease after ambient AI in the mean score for mental demand (−6.12 [95% CI; −7.52 to −4.72]; *P* < .001), hurried or rushed pace (−6.96 [95% CI, −8.42 to −5.50]; *P* < .001), and effort to accomplish note writing (−5.57 [95% CI, −6.93 to −4.21]; *P* < .001). No respondent characteristics were associated with those outcomes. Logistic regression indicated that medical and surgical subspecialties were less likely to agree or strongly agree that Abridge increased their work satisfaction compared with primary care (odds ratio, 0.02 [95% CI, <0.01 to 0.16]) (eTable 3 in Supplement 1). Multivariate linear modeling also indicated that respondents in medical or surgical subspecialties had lower mean ratings in overall experience (−1.35 [95% CI, −2.46 to −0.23]; *P* = .02) and recommend ambient AI (−2.03 [95% CI, −3.2 to −0.85]; *P* < .001) scores compared with respondents in primary care (eTable 3 in Supplement 1).

### Open-Ended Survey Questions

Open-ended survey responses reinforced and expanded on quantitative findings (Table 3). Overall, benefits from ambient AI included improved comprehensiveness of notes, improved efficiency, and improved visit experience. Among the benefits, clinicians frequently noted that ambient AI had reduced their cognitive load and the general burden of documentation. Many of these clinicians reported that they felt this technology should be immediately available to all clinicians.

**Table 3.** Main Themes and Example Quotations From Open-Ended Postsurvey Comments

Theme	Example quotation
**Benefits of ambient AI**	
Improved comprehensiveness of notes	“The Patient Visit Summary is incredibly useful given the level of detail. And also being able to respond urgently to high priority issues and know that my notes are captured and saved even if I am not able to address the chart that day.”
More connection with patients during visit	“I have loved my experience with Abridge thus far. I am so incredibly pleased with the ease of use and the quality of the notes. I can devote much more face-to-face, quality time with patients and less time staring at the computer, typing, or taking hand-written notes. I do not feel nearly as stressed about the burden of note-writing, especially with complicated patients.”
Reduced cognitive load	“Abridge has reduced mental stress. I could provide personal attention to patient more.”
Increased well-being	“I go home happy. It’s brin[g]ing back the joy of medicine–taking care of patients without the drain of doing clerical work.”
Share with colleagues	“We have got to spread this to our colleagues sooner rather than later.”
**Challenges with ambient AI**	
Not integrated in the EHR	“…having [to] go from the phone, to Abridge to Epic is too much. I want to edit in just one place.”
Unable to customize or format progress note	“It would be amazing if Abridge can learn provider writing style and can be updated/customized accordingly.”
Specialty specific issues with generated content	“The physical exam part is not used at all because it is not tailored to a specialist’s exam, which in my situation is the Neurological exam.”
Additional functionalities like dictation	“I would like for it to have a dictation part so I do not have to use another 3rd party software to dictate my assessment and plan.”
Potential for improvement in the future	“I am very committed to making this work, and I really believe that AI will be the way we chart in the future, so I want to stick with it as improvements are made.”

Abbreviations: AI, artificial intelligence; EHR, electronic health record.

Nevertheless, there were still many respondents who reported challenges with ambient AI, primarily because the AI technology was not fully embedded within the EHR at the time the pilot project launched. Other respondents wanted additional functionality, such as the ability to turn on a “direct dictation” mode or have AI integrate with orders. Another reported challenge was that the generated note did not account for different specialties, particularly with the physical exam. Despite challenges, a few participants mentioned the “potential” they saw for ambient AI, with some individuals expressing a willingness to handle some more challenging aspects of the ambient AI now because they felt it would be beneficial in the future.

### EHR Metrics

Of 100 participants, 92 individuals had EHR metrics available 3 months before and after ambient AI implementation (Table 4). Mean (SD) time in notes per appointment significantly decreased, from 6.2 (4.0) to 5.3 (3.5) minutes (*P* < .001) after ambient AI. Although the time spent in notes decreased, the documentation length by mean (SD) number of characters increased from 4326 (2328) to 4548 (2226) characters (*P* = .01) and mean (SD) progress note length increased from 5683 (2791) to 5961 (2781) characters (*P* < .001). Mean time in (SD) off-hour EHR activities was similar before and after ambient AI implementation (38.2 [42.0] vs 39.8 [42.9 ] minutes; *P* = .14). Subgroup DiD analyses indicated that while both males and females had decreases, females had a bigger decrease for mean (SD) time in notes per appointment of 1.4 minutes (8.1 [3.9] to 6.7 [3.6] minutes) vs 0.5 minutes for males (4.7 [3.5] to 4.2 [3.1] minutes) (*P* = .001). Females also had a significant increase in mean (SD) document length (4197 [2850] to 4529 [2743] characters; *P* = .001) and progress note length (5821 [3145] to 6175 [3048] characters; *P* < .001) (Table 4). Primary care and medical subspecialties had increased mean (SD) progress note length (5035 [1406] to 5383 [1288] characters; *P* = .005 and 8502 [3923] to 8724 [3815] characters; *P* = .02, respectively) and decreased mean (SD) time in notes (6.3 [3.0] to 5.2 [2.7] minutes; *P* < .001 and 9.5 [5.2] to 8.4 [4.3] minutes; *P* = .03) (Table 5). After Bonferroni adjustment (α = 0.05/20), most outcomes kept the same statistical significance; however, progress note length and time in notes per appointment for medical subspecialties became nonsignificant.

**Table 4.** EHR Clinician Metrics Overall and by Sex^a^

Metric	Overall				Male				Female				DiD *P* value^b^
	Clinicians, No.	Pre	Post	*P* value	Clinicians, No.	Pre	Post	*P* value	Clinicians, No.	Pre	Post	*P* value	
Documentation length, characters													
Mean (SD)^c^	92	4326 (2328)	4548 (2226)	.01	51	4431 (1828)	4564 (1733)	.33	41	4197 (2850)	4529 (2743)	.001	.25
Median (IQR)^d^	92	4012 (3004-5351)	4081 (3344-5394)	<.001	51	4209 (3069-5503)	4085 (3472-5335)	.02	41	3895 (2801-5117)	4076 (3191-5391)	<.001	
Progress note length, characters													
Mean (SD)^c^	92	5683 (2791)	5961 (2781)	<.001	51	5572 (2496)	5790 (2564)	.08	41	5821 (3145)	6175 (3048)	<.001	.38
Median (IQR)^d^	92	4991 (3930-6728)	5472 (4297-6896)	<.001	51	4580 (3797-6862)	5146 (4117-7092)	.004	41	5167 (4171-6536)	5662 (4468-6771)	<.001	
Time in notes per appointment, min													
Mean (SD)^c^	91	6.2 (4.0)	5.3 (3.5)	<.001	51	4.7 (3.5)	4.2 (3.1)	.004	40	8.1 (3.9)	6.7 (3.6)	<.001	.001
Median (IQR)^d^	91	5.5 (3.2-7.8)	4.6 (2.6-7)	<.001	51	3.8 (2-6.5)	3.1 (2-5.2)	.004	40	7.6 (5.5-10.2)	6 (4.4-8.7)	<.001	
Off-hour EHR activities, min^e^													
Mean (SD)^c^	91	38.2 (42.0)	39.8 (42.9)	.14	51	30.4 (35.1)	32 (36.1)	.13	40	48.2 (48.1)	49.7 (48.9)	.46	.95
Median (IQR)^d^	91	24.1 (9.6-51.1)	25.9 (8.8-51.2)	.21	51	19.7 (7.7-45)	24.7 (6.4-42.1)	.13	40	29.6 (14.4-78.6)	29.5 (14.3-85.8)	.71	

Abbreviations: DiD, difference in difference; EHR, electronic health record.

^a^ Metrics are given for preimplementation and postimplementation periods (3 months before and after implementation of ambient listening, respectively).

^b^ DiD is for male vs female.

^c^ Paired *t* test.

^d^ Wilcoxon signed-rank test.

^e^ Refers to EHR work 5:30 pm to 7:00 am on weekdays and nonscheduled weekends and holidays.

**Table 5.** EHR Clinician Metrics by Specialty^a^

Metric	Primary care				Medical subspecialties				DiD 1, *P* value^b^	Surgical subspecialties				DiD 2 *P* value^b^
	Clinicians, No.	Pre	Post	*P* value	Clinicians, No.	Pre	Post	*P* value		Clinicians, No.	Pre	Post	*P* value	
Documentation length, characters														
Mean (SD)^c^	54	3731 (1539)	3987 (1358)	.04	20	6253 (3518)	6586 (3288)	.13	.72	18	3971 (1477)	3967 (1569)	.96	.25
Median (IQR)^d^	54	3417 (2719-4618)	3819 (3131-4863)	<.001	20	5377 (4447-6754)	5731 (4547-7203)	.02		18	3754 (2980-4649)	3932 (2961-4267)	.97	
Progress note length, characters														
Mean (SD)^c^	54	5035 (1406)	5383 (1288)	.005	20	8502 (3923)	8724 (3815)	.02	.52	18	4494 (2476)	4627 (2835)	.33	.29
Median (IQR)^d^	54	4918 (3942-6003)	5348 (4380-6054)	<.001	20	7031 (6008-10168)	7362 (6200-10550)	.001		18	4016 (2946-4718)	3966 (3005-4959)	.70	
Time in notes per appointment, min														
Mean (SD)^c^	54	6.3 (3.0)	5.2 (2.7)	<.001	19	9.5 (5.2)	8.4 (4.3)	.03	.98	18	2.3 (1.6)	2.3 (1.7)	>.99	.002
Median (IQR)^d^	54	5.7 (4.1-7.8)	4.8 (3-6.6)	<.001	19	7.6 (5.5-14.1)	8 (5.2-11.3)	.04		18	2 (1.1-3.1)	2 (1.2-2.7)	.72	
Off-hour EHR activities^e^														
Mean (SD)^c^	54	37.4 (42.1)	39.5 (44.7)	.16	19	44.3 (35.5)	46.7 (35.3)	.33	.93	18	34.1 (49.3)	33.3 (45.5)	.69	.31
Median (IQR)^d^	54	24.4 (8.2-47.4)	25.3 (8.8-49)	.36	19	34.4 (19-64.8)	30.2 (20-74.4)	.28		18	14.1 (9.7-41.6)	13.4 (6.4-44.3)	>.99	

Abbreviation: DiD, difference in difference; EHR, electronic health record.

^a^ Metrics are given for preimplementation and postimplementation periods (3 months before and after implementation of ambient listening, respectively).

^b^ DiD 1 is for primary care vs medical subspecialties, and DiD 2 is for primary care vs surgical subspecialities.

^c^ Paired *t* test.

^d^ Wilcoxon signed-rank test.

^e^ Refers to EHR work 5:30 PM to 7:00 AM on weekdays and nonscheduled weekends and holidays.

Linear mixed modeling of EHR metrics (eTable 4 in Supplement 1) showed a mean increase of 210.66 characters (95% CI, 28.18 to 393.15 characters) in documentation length (*P* = .02) and 258.82 characters (95% CI, 97.00 to 420.64 characters) in progress note length (*P* = .002) after ambient AI. Time in notes per appointment was reduced by less than 1 minute (0.91 minutes [95% CI, −1.20 to −0.62 minutes]; *P* < .001), but off-hour EHR activities was not significantly changed. Medical subspecialties had a mean increase of 2323.72 characters (95% CI, 994.70 to 3652.73 characters; *P* = .001) in document length and 3.75 minutes (95% CI, 2.09 to 5.41 minutes; *P* < .001) in notes per appointment compared with primary care. Surgical subspecialties spent a mean of 2.45 minutes (95% CI, −4.10 to −0.81 minutes) less in notes per appointment than primary care (*P* = .004). Compared with male clinicians, female clinicians spent a mean of 1.97 more minutes (95% CI, 0.71 to 3.22 minutes) in notes per appointment (*P* = .002).

## Discussion

This quality improvement study is one of the first to investigate the association of ambient AI document implementation with clinician outcomes by combining quantitative and qualitative data from various sources: surveys, open-ended comment thematic analysis, and EHR metrics. Overall, our pilot project found that ambient AI was associated with decreased cognitive load (mental demand, temporal demand, and effort) like Shah et al^10^ found and with improved satisfaction at work, echoing previous research,^10,15^ although our respondents reported higher levels of satisfaction.^9^ While burnout decreased overall after ambient AI implementation, this change was not significant, differing from previous findings. Given the current climate of depersonalization of medicine^16^ and overall dissatisfaction with work,^17,18^ our findings suggest that ambient AI could serve as a valuable means of renewing focus on the clinician-patient interaction rather than the burden of documentation, thus improving the experience for all.

To date, studies of ambient AI have shown varied results for the association with time in notes, off-hour EHR activities, and after-hours work.^7,11,12^ While our clinicians had a significant decrease in time spent in notes per appointment, they did not see an association with off-hour EHR activities or other after-hours work. Flexibility and control over schedule have been suggested to be important components of clinician well-being,^19^ and it could be that ambient AI provides clinicians with needed flexibility to choose when to complete their notes.

Interestingly, progress notes and documentation length were longer after use of ambient AI, which is similar to previous findings.^12^ As noted by our survey respondents, this may be because ambient AI helps to make their progress notes more comprehensive and detailed. We found that even with ambient AI, females in our pilot had longer progress notes and documentation length than males, which is similar to a study in which female surgeons had longer progress notes than their male counterparts.^20^ Female clinicians have also been shown to have longer patient visits,^21,22^ which may be 1 reason their progress note lengths are generally longer.

More primary care clinicians reported that ambient AI improved their overall satisfaction at work (85.8%) compared with clinicians in medical (36.4%) and surgical (50.0%) subspecialties. As one of the themes from the open-ended commented, this may in part be because ambient AI currently does not generate documentation accounting for needs of each specialty like with physical exam results. Regarding time spent in notes per appointment, medical subspecialties had the highest mean time, followed by primary care and then surgical subspecialties. In fact, we found that medical subspecialties had an increase in time spent in notes per appointment and had the longest progress notes, even after controlling for other factors. While this differs from previous study results,^23^ one theme suggested that this may be due to currently being unable to make a more permanent customization or formatting of specific sections of the progress note. Specialists may still need to manually add these sections to the progress note after ambient AI generation, which may require time and increase total length.^24^

In survey comments, clinicians mentioned several challenges. However, given the rapidly evolving nature of generative AI, many issues mentioned by respondents have already been or are in the process of being corrected. For example, ambient AI has since been fully integrated into the Sutter EHR, and we are in the process of collecting data to understand experiences of clinicians since this change. Some comments also suggested that clinicians recognized the great potential and value that may come from this technology, which is similar to what others have suggested: “AI will improve its outcomes, efficiency, and hopefully, sustainability as a career path. These AI solutions offer great potential benefit.”^25^

### Limitations

This study has several limitations. These findings are from a single organization and so may not be generalizable. Given that this was a quality improvement pilot project, we could make real-time adjustments to benefit clinical implementation, including repurposing additional licenses to replace clinicians who declined participation, which introduced potential bias. We purposively sampled clinicians to ensure adequate representation from regions and specialties and to include more clinical leaders and informaticists to provide initial feedback that could be incorporated into subsequent launches. While this is beneficial in quality improvement, there was potential bias given that experiences may not be representative. While preintervention and postintervention surveys were offered multiple times, there may have been response bias. Nevertheless, we have a higher completion rate for both surveys compared with another evaluation of similar technology.^8^ The relatively small sample size for medical and surgical subspecialties in survey responses may not be representative; a larger sample in the future may be needed for validation. It is possible that 3 months after implementation is insufficient time for some metrics, but other evaluations have used a shorter time (eg, 10 weeks),^11^ and so we are in line with previous work.

## Conclusions

This quality improvement study pilot evaluation found that ambient AI was associated with improved overall well-being for many clinicians through decreased mental demand of documentation, improved connection with patients, and decreased amount of time in notes, both self-reported and as evidenced by EHR metrics. In its current version, ambient AI may have had varying outcomes for clinicians by sex and specialty. Future research is needed to understand specific experiences of these subgroups and overall outcomes after widescale expansion given the context of a rapidly evolving technology, which shows promise in its ability to improve the joy of work for clinicians and their ability to connect with patients.

## Notes

Supplement 1. **eTable 1.** Postsurvey Results **eTable 2.** Linear Mixed-Effect Models for Outcomes in Both Surveys **eTable 3.** Logistic Regression and Linear Regression for Postsurvey Outcomes **eTable 4.** Linear Mixed-Effect Models for EHR Signal Outcomes

Supplement 2.
Data Sharing Statement
