# Supplementary material for: Evaluation of an Ambient Artificial Intelligence Documentation Platform for Clinicians
Source: JAMA Netw Open. 2025 May 2;8(5):e258614. doi: 10.1001/jamanetworkopen.2025.8614 (PMC12048851; doi:10.1001/jamanetworkopen.2025.8614)
Supplement: Supplement 1. — eTable 1. Postsurvey Results eTable 2. Linear Mixed-Effect Models for Outcomes in Both Surveys eTable 3. Logistic Regression and Linear Regression for Postsurvey Outcomes eTable 4. Linear Mixed-Effect Models for EHR Signal Outcomes [file jamanetwopen-e258614-s001.pdf]

Supplemental Online Content

Stults CD, Deng S, Martinez MC, et al. Evaluation of an ambient artificial intelligence documentation platform for clinicians. *JAMA Netw Open*. 2025;8(5):e258614. doi:10.1001/jamanetworkopen.2025.8614

eTable 1. Postsurvey Results

eTable 2. Linear Mixed-Effect Models for Outcomes in Both Surveys

eTable 3. Logistic Regression and Linear Regression for Postsurvey Outcomes

eTable 4. Linear Mixed-Effect Models for EHR Signal Outcomes

This supplemental material has been provided by the authors to give readers additional information about their work.

© 2025 Stults CD et al. *JAMA Network Open*.

eTable 1. Postsurvey Results

Variable	Overall (n=57)	Male (n=31)	Female (n=26)	*P* value	Primary Care (n=38)	Medical subspecialty (n=11)	Surgical subspecialty (n=8)	*P* value
**Work Satisfaction (agree/strongly agree), No. (%)**[a]	41 (71.9)	22 (71.0)	19 (73.1)	>.99	33 (85.8)	4 (36.4)	4 (50.0)	<.001
**Overall Abridge Experience**								
Mean (SD)[b]	7.8 (1.8)	7.8 (2.0)	7.9 (1.7)	.82	8.2 (1.1)	6.6 (2.6)	7.6 (2.6)	.04
Median (IQR)[c]	8 (7-9)	8 (7.5-9)	8 (7-9)	.88	8 (7-9)	7 (5-8.5)	8 (7.5-9.25)	.68
**Recommend Abridge**								
Mean (SD)[b]	8.5 (2.1)	8.4 (2.5)	8.6 (1.5)	.77	9.1 (1.1)	6.9 (2.9)	7.8 (3.1)	.003
Median (IQR)[c]	9 (8-10)	10 (7.5-10)	9 (8-10)	.42	9.5 (8-10)	7 (5-9.5)	9.5 (6-10)	.40

a. X^2 test or Fisher exact test
b. 1-way analysis of variance
c. Moon median test

© 2025 Stults CD et al. *JAMA Network Open*.

eTable 2. Linear Mixed-Effect Models for Outcomes in Both Surveys

	Mental Demand		Temporal Demand		Effort		mini-Z burnout		Undivided Attention		Note writing outside work	
Variables	Coefficient (95% CI)	*P* value	Coefficient (95% CI)	*P* value	Coefficient (95% CI)	*P* value	Odds Ratio (95% CI)	*P* value	Odds Ratio (95% CI)	*P* value	Odds Ratio (95% CI)	*P* value
**Survey**												
Preimplementation	[Reference]		[Reference]		[Reference]		[Reference]		[Reference]		[Reference]	
Postimplementation	-6.12 (-7.52 to 4.72)	<0.001	-6.96 (-8.42 to -5.50)	<.001	-5.57 (-6.93 to -4.21)	<.001	0.7 (0.21 to 2.28)	.55	14.33 (3.62 to 56.70)	<.001	15.15 (4.35 to 52.74)	<.001
**Age**	0.06 (-0.05 to 0.17)	.30	0.01 (-0.11 to 0.13)	.88	0.04 (-0.09 to 0.16)	.56	0.94 (0.81 to 1.09)	.39	1.07 (0.99 to 1.16)	.07	0.92 (0.83 to 1.02)	.12
**Sex**												
Male	[Reference]		[Reference]		[Reference]		[Reference]		[Reference]		[Reference]	
Female	1.31 (-0.40 to 3.03)	.13	0.14 (-1.76 to 2.04)	.88	1.61 (-0.29 to 3.51)	.10	6.88 (0.81 to 58.32)	.08	0.98 (0.32 to 2.97)	.97	0.12 (0.02 to 0.57)	.01
**FTE**												
>=0.7	[Reference]		[Reference]		[Reference]		[Reference]		[Reference]		[Reference]	
0.5 to <0.7	-1.21 (-3.35 to 0.94)	.27	0.36 (-2.02 to 2.74)	.77	0.47 (-1.91 to 2.85)	.70	0.3 (0.02 to 4.02)	.36	0.27 (0.06 to 1.15)	.08	1.87 (0.28 to 12.73)	.52
<0.5	-2.4 (-5.19 to 0.40)	.09	0.75 (-2.35 to 3.85)	.63	-0.85 (-3.95 to 2.25)	.59	1.52 (0.05 to 43.24)	.81	0.14 (0.02 to 0.93)	.04	2.58 (0.23 to 29.35)	.45
**Number of years of service**	0.01 (-0.14 to 0.13)	.97	-0.05 (-0.20 to 0.10)	.54	0.03 (-0.12 to 0.19)	.65	0.98 (0.82 to 1.17)	.82	1.01 (0.92 to 1.11)	.76	1.04 (0.92 to 1.17)	.51
**Specialty group**												
Primary Care	[Reference]		[Reference]		[Reference]		[Reference]		[Reference]		[Reference]	
Medical or Surgical Subspecialty	-0.07 (-1.92 to 1.78)	.94	-0.73 (-2.78 to 1.32)	.48	1.11 (-0.94 to 3.17)	.29	1.14 (0.12 to 10.56)	.91	1.88 (0.55 to 6.46)	.31	1.29 (0.25 to 6.68)	.76

© 2025 Stults CD et al. *JAMA Network Open*.

eTable 3. Logistic Regression and Linear Regression for Postsurvey Outcomes

	**Work Satisfaction**		**Overall Abridge Experience**		**Recommend Abridge**	
Variables	Odds Ratio (95% CI)	*P* value	Coefficient (95% CI)	*P* value	Coefficient (95% CI)	*P* value
**Age**	1.06 (0.93 to 1.23)	.45	0.01 (-0.05 to 0.08)	.71	0.03 (-0.04 to 0.1)	.37
**Sex**						
Male	[Reference]		[Reference]		[Reference]	
Female	0.54 (0.07 to 3.31)	.52	-0.03 (-1.06 to 1.01)	.96	0.004 (-1.08 to 1.09)	.99
**FTE**						
>=0.7	[Reference]		[Reference]		[Reference]	
0.5 to <0.7	0.78 (0.08 to 6.70)	.82	0.06 (-1.24 to 1.36)	.93	-0.02 (-1.39 to 1.34)	.97
<0.5	3.09 (0.21 to 66.69)	.43	0.26 (-1.43 to 1.95)	.76	-0.32 (-2.09 to 1.46)	.72
**Number of years of service**	0.82 (0.65 to 0.97)	.05	-0.06 (-0.14 to 0.02)	.14	-0.07 (-0.15 to 0.02)	.12
**Specialty group**						
Primary Care	[Reference]		[Reference]		[Reference]	
Medical or Surgical Subspecialty	0.021 (0.001 to 0.16)	.002	-1.35 (-2.46 to -0.23)	.02	-2.03 (-3.2 to -0.85)	.001

© 2025 Stults CD et al. *JAMA Network Open*.

eTable 4. Linear Mixed-Effect Models for EHR Signal Outcomes

	Documentation Length		Progress Note Length		Time in Notes per Appointment		Off-hour EHR Activities	
**Predictors**	Regression Coefficient (95% CI)	*P* value	Regression Coefficient (95% CI)	*P* value	Regression Coefficient (95% CI)	*P* value	Regression Coefficient (95% CI)	P value
**Ambient AI Training**								
Preimplementation	[Reference]		[Reference]		[Reference]		[Reference]	
Postimplementation	210.66 (28.18 to 393.15)	.02	258.82 (97.00 to 420.64)	.002	-0.91 (-1.20 to -0.62)	<.001	0.75 (-1.28 to 2.79)	.47
**Age**	50.96 (-18.22 to 120.13)	.15	36.26 (-44.88 to 117.39)	.38	0.01 (-0.08 to 0.09)	.85	0.03 (-1.15 to 1.21)	.96
**Sex**								
Male	[Reference]		[Reference]		[Reference]		[Reference]	
Female	-213.55 (-1231.91 to 804.81)	.68	88.49 (-1105.88 to 1282.87)	.89	1.97 (0.71 to 3.22)	.002	3.48 (-13.61 to 20.56)	.69
**Race**								
White	[Reference]		[Reference]		[Reference]		[Reference]	
Asian	142.66 (-1112.23 to 1397.55)	.82	509.28 (-962.50 to 1981.06)	.50	-0.43 (-1.98 to 1.12)	.59	-11.36 (-32.45 to 9.72)	.29
Multiple races/Unknown	602.09 (-1534.12 to 2738.30)	.58	889.17 (-1616.25 to 3394.59)	.49	-0.06 (-2.69 to 2.57)	.97	-17.72 (-53.45 to 18.02)	.33
**Ethnicity**								
Non-Hispanic	[Reference]		[Reference]		[Reference]		[Reference]	
Hispanic	-859.91 (-2688.34 to 968.52)	.36	-753.28 (-2897.73 to 1391.17)	.49	2.15 (-0.11 to 4.41)	.06	12.32 (-18.36 to 43.00)	.43
Unknown	-835.05 (-3448.03 to 1777.93)	.53	538.64 (-2525.96 to 3603.23)	.73	3.64 (0.42 to 6.85)	.03	7.14 (-36.57 to 50.85)	.75
**No. of years of service**	-71.51 (-160.30 to 17.27)	.11	-39.57 (-143.70 to 64.56)	.46	-0.03 (-0.14 to 0.08)	.61	0.25 (-1.24 to 1.74)	.74
**FTE**								
>=0.7	[Reference]		[Reference]		[Reference]		[Reference]	
0.5 to <0.7	426.14 (-814.05 to 1666.33)	.50	469.7 (-984.83 to 1924.24)	.53	0.82 (-0.72 to 2.37)	.30	12.7 (-8.32 to 33.72)	.24
<0.5	-435.36 (-2084.04 to 1213.32)	.61	-312.11 (-2245.73 to 1621.52)	.75	0.85 (-1.19 to 2.89)	.42	-7.43 (-35.16 to 20.31)	.60
**Specialty Grouping**								
Primary Care	[Reference]		[Reference]		[Reference]		[Reference]	

© 2025 Stults CD et al. *JAMA Network Open*.

Medical Subspecialty	2323.72 (994.70 to 3652.73)	.001	3407.99 (1849.27 to 4966.71)	<.001	3.75 (2.09 to 5.41)	<.001	15.12 (-7.43 to 37.66)	.19
Surgical Subspecialty	175.19 (-1162.88 to 1513.27)	.80	-65.46 (-1634.80 to 1503.89)	.94	-2.45 (-4.10 to -0.81)	.004	-4.31 (-26.69 to 18.08)	.71

© 2025 Stults CD et al. *JAMA Network Open*.
