# Supplementary material for: Evaluation of an Ambient Artificial Intelligence Documentation Platform for Clinicians
Source: JAMA Netw Open. 2025 May 2;8(5):e258614. doi: 10.1001/jamanetworkopen.2025.8614 (PMC12048851; doi:10.1001/jamanetworkopen.2025.8614)
Supplement: Supplement 2. — Data Sharing Statement [file jamanetwopen-e258614-s002.pdf]

Data Sharing Statement

Stults. Evaluation of an Ambient Artificial Intelligence Documentation Platform for Clinicians. *JAMA Netw Open*. Published online May 2, 2025. doi:10.1001/jamanetworkopen.2025.8614

Data
Data available: No
